# Complete Genome Sequences of 12 Isolates of *Listeria monocytogenes* Belonging to Serotypes 1/2a, 1/2b, and 4b Obtained from Food Products and Food-Processing Environments in Canada

**DOI:** 10.1128/genomeA.00258-17

**Published:** 2017-05-11

**Authors:** Walid Mottawea, Shu Chen, Saleema Saleh-Lakha, Sebastien Belanger, Dele Ogunremi

**Affiliations:** a Department of Food Science and Agricultural Chemistry, Faculty of Agricultural and Environmental Sciences, McGill University, Montreal, Quebec, Canada; b Department of Microbiology and Immunology, Faculty of Pharmacy, Mansoura University, Mansoura, Egypt; c Laboratory Services Division, University of Guelph, Guelph, Ontario, Canada; d Ottawa Laboratory Fallowfield, Canadian Food Inspection Agency, Ottawa, Ontario, Canada

## Abstract

*Listeria monocytogenes* is the etiological agent for an often fatal foodborne illness known as listeriosis. Here, we present the complete genome sequences of 12 *L. monocytogenes* isolates representing the three most common serotypes of this pathogen (1/2a, 1/2b, and 4b), collected in Canada from different food products and environmental sources.

## GENOME ANNOUNCEMENT

Listeriosis, a life-threatening infection caused by* Listeria monocytogenes*, results in one of the highest mortality rates among foodborne illnesses in Canada, with an average of 44 deaths each year (90% probability intervals: 31-76 deaths) (1). In order to reduce the risk of listeriosis, studies aimed at understanding the nature and behavior of the causative bacterial organism at the genomic level are necessary. *L. monocytogenes* is a ubiquitous, Gram-positive bacterium commonly found in natural environments such as plants, soil, and surface water, from where it contaminates agricultural products, food-processing environments, and foods such as fresh produce and ready-to-eat meat and milk, leading to possible human exposure (2). More than 95% of human clinical cases are associated with three serotypes of *L. monocytogenes*: 1/2a, 1/2b, and 4b (3). Here, we report the complete genome sequences of 12 *L. monocytogenes* isolates belonging to these serotypes. The strains were collected from foods and food-processing environments in Canada during the period from 2002 to 2009 as part of routine testing procedures carried out by the Agriculture and Food Laboratory in Guelph, Ontario, Canada. Strains were selected to represent both predominant and unique genotypes among over 2,400 strains tested during a comprehensive genotypic analysis (4).

Genomic DNA samples were extracted from overnight cultures of the 12* L. monocytogenes* strains using the Wizard Genomic DNA purification Kit (Promega, Madison, WI, USA). Each isolate was sequenced using PacBio (Pacific BioSciences Inc., Menlo Park, CA, USA) and Illumina MiSeq (Illumina Inc., San Diego, CA, USA) technologies, as per standard protocols in 2013, and assembled independently. The PacBio reads were assembled with the Celera assembler using the HGAP/Quiver protocol (5) and corrected with Illumina sequence data. The Illumina reads, on the other hand, were assembled using the hybrid approach as previously described (6). The final corrected genome for each chromosome was developed by aligning an *in silico* map of the Illumina read-corrected PacBio assembly with the map of the corresponding, independently assembled Illumina sequence against an optical, *de novo* whole-genome map. The map consisted of *NheI* restriction sites and was developed using the Argus optical mapping system (OpGen Inc., Gaithersburg, MD, USA), and the analysis was done using the MapSolver software. The nucleotide sequences of all detected gaps and misalignments were corrected using Clone Manager software (Professional edition, Scientific and Educational Software, Cary, NC, USA).

The generated genomes range from 2,913,947 to 3,072,093 bp with a GC content of 37.95 ± 0.02% (average ± standard error of the mean [SEM]). We used the Rapid Annotations using Subsystem Technology server (7– 9) to annotate each genome. We identified 2,944 ± 21.6 (average ± SEM) protein-coding sequences and 64 ± 1.29 (average ± SEM) tRNAs per genome. Alignment of all 12 genome sequences using the Mauve system of multiple genome alignment (10) demonstrated clustering based on serotypes. Average nucleotide identity analysis (http://enve-omics.ce.gatech.edu/ani) confirmed similarities within each serotype (1/2a: 98.60 to 98.99%, *n* = 5; 1/2b: 99.44 to 99.94%, *n* = 3; 4b: 99.47 to 99.60%, *n* = 4) and was able to differentiate between serotype 1/2a and the other two serotypes (versus 1/2b: 94.32 to 94.47%; versus 4b: 94.19 to 94.35%); however, serotypes 1/2b and 4b were very similar (99.42 to 99.57%). A complete comparative genomic study of the 12 strains will be presented elsewhere.

### Accession number(s).

The complete genome sequences of these 12 *L. monocytogenes* isolates were deposited in GenBank under BioProject no. PRJNA 371291. Accession numbers are shown for each isolate in Table 1 (CP019614 to CP019625).

**TABLE 1:** GenBank accession numbers of 12 *L. monocytogenes* isolates from food products and food-processing environments

Isolate	Identification source	Serotype	GenBank accession no.
10-092876-1559	Chicken breast	1/2a	CP019614
10-092876-0168	Sprouts	1/2b	CP019615
10-092876-1063	Baby spinach	4b	CP019616
10-092876-0055	Environmental swab	1/2a	CP019617
10-092876-0731	Environmental sponge	1/2a	CP019618
10-092876-1155	Environmental swab	4b	CP019619
10-092876-1547	Environmental swab	4b	CP019620
10-092876-1235	Baby spinach	1/2a	CP019621
10-092876-0145	Ground beef	1/2b	CP019622
10-092876-1763	Chicken breast	1/2a	CP019623
10-092876-1016	Ready-to-eat fermented meat	1/2b	CP019624
10-092876-0769	Environmental sponge	4b	CP019625
